# *In Vivo* Imaging-Based Mathematical Modeling Techniques That Enhance the Understanding of Oncogene Addiction in relation to Tumor Growth

**DOI:** 10.1155/2013/802512

**Published:** 2013-03-20

**Authors:** Chinyere Nwabugwu, Kavya Rakhra, Dean Felsher, David Paik

**Affiliations:** ^1^Department of Electrical Engineering, Stanford University School of Medicine, Stanford, CA 94305, USA; ^2^Department of Radiology, Stanford University School of Medicine, Stanford, CA 94305, USA; ^3^Department of Medicine, Stanford University School of Medicine, Stanford, CA 94305, USA

## Abstract

The dependence on the overexpression of a single oncogene constitutes an exploitable weakness for molecular targeted therapy. These drugs can produce dramatic tumor regression by targeting the driving oncogene, but relapse often follows. Understanding the complex interactions of the tumor's multifaceted response to oncogene inactivation is key to tumor regression. It has become clear that a collection of cellular responses lead to regression and that immune-mediated steps are vital to preventing relapse. Our integrative mathematical model includes a variety of cellular response mechanisms of tumors to oncogene inactivation. It allows for correct predictions of the time course of events following oncogene inactivation and their impact on tumor burden. A number of aspects of our mathematical model have proven to be necessary for recapitulating our experimental results. These include a number of heterogeneous tumor cell states since cells following different cellular programs have vastly different fates. Stochastic transitions between these states are necessary to capture the effect of escape from oncogene addiction (i.e., resistance). Finally, delay differential equations were used to accurately model the tumor growth kinetics that we have observed. We use this to model oncogene addiction in MYC-induced lymphoma, osteosarcoma, and hepatocellular carcinoma.

## 1. Introduction

Bernard Weinstein first proposed in 1997 that “oncogene addiction” is the phenomenon whereby the inactivation of a single oncogene, even if brief, may lead to sustained tumor regression, providing a weakness for a molecularly targeted therapy to exploit [1]. For example, imatinib causes dramatic tumor regression in gastrointestinal stromal tumors (GIST) [2, 3] and chronic myelogenous leukemia (CML) [3–5] by inhibiting the *Bcr-Abl* oncogene; erlotinib and gefitinib cause dramatic tumor regression in nonsmall cell lung cancer (NSCLC) [6–9], pancreatic cancer, and other tumors by inhibiting EGFR; a number of other examples of targeted therapies exist. These drugs induce dramatic tumor regression without the side effect profile of nonspecific chemotherapies.

Inactivation of the oncogene by targeted therapy produces a complex array of responses at the cellular level including apoptosis, cell cycle arrest, differentiation, senescence, and inhibition of angiogenesis. In preclinical models, the oncogene may be inactivated using conditional expression in transgenic animals (e.g., Cre/LoxP, tamoxifen, or tetracycline systems). Some of these resultant cellular programs are cell intrinsic (i.e., not involving other cells) while others are cell extrinsic, involving complex host interactions with effector cells in the immune system. While these different response mechanisms have been studied and modeled individually, there has been far less investigation into integrating the overall sequence and interactions of tumor responses into a unified mathematical model that can inform the design and optimization of therapeutic strategies. Understanding how and why some tumors relapse while others do not, as well as how and why the specific cellular program responses depend on the tissue-specific and host immune background, is of crucial importance for designing the most effective therapies.

Previously, we have built and validated a model of tumor growth and regression kinetics in response to oncogene inactivation [10]. This model was based primarily upon microCT imaging and immunohistochemistry (IHC) and explicitly incorporated apoptosis and proliferation resulting from the stochastic balance between prosurvival and prodeath signals but included no other cellular programs. In other work, we have empirically shown the importance of cellular senescence, immune surveillance, differentiation, and angiogenesis. Here, we have created a mathematical model that now captures the tumor growth kinetics as a function of all of the aforementioned cellular programs informed primarily by bioluminescence imaging (BLI) and IHC. We are building on this to develop and calibrate a novel integrative mathematical model of the tumor responses to oncogene inactivation (cell intrinsic and cell extrinsic) that is designed to eventually predict, optimize, and validate various therapeutic strategies.

We will use the model to study the major cellular processes involved in MYC-induced lymphoma, osteosarcoma, and hepatocellular carcinoma, which involve difference combinations and sequences of these programs and to test different therapeutic strategies.

Much work has been done in characterizing tumor growth kinetics *in vivo *and in mathematically modeling the cell intrinsic mechanisms involved in the response to oncogene inactivation. *In vivo *observations of cell extrinsic mechanisms in response to oncogene inactivation have been published recently, but little if any mathematical or computational modeling has been done to complement these theories. Our work is among the first to simultaneously model all of the complex immune-mediated responses that are critical in determining the factors involved in tumor relapse thereby providing understanding of how to prevent it.

## 2. Materials and Methods

### 2.1. Biological Data

We utilize the tetracycline (Tet) system to conditionally and reversibly control the expression of the MYC oncogene in mouse models [11, 12]. Even in the absence of a putative drug, this models the effect of a targeted therapeutic that would downregulate the aberrant overexpression of MYC as a treatment for the tumor. In the Tet-Off system, doxycycline (dox) is added to the drinking water to inhibit binding of the tet-transactivating promoter (tTA) to the Tet-O promoter and thus inactivates transcription of MYC. Alternatively, in the Tet-On system, dox allows binding of reverse tTA (rtTA) and thus activates transcription of MYC [13]. MYC expression can even be titrated with a threshold on tumor regression occurring at ≤0.05 *μ*g/mL of dox (in ≤0.2 ng/mL plasma concentration) in a Tet-On system [14]. We have collected data from various conditional mouse models of MYC and concentrated on the tumor type specific responses to MYC inactivation seen in various tumor types including lymphoma (apoptosis, proliferative arrest, differentiation, senescence, antiangiogenesis, and tumor relapse) [15–17], osteosarcoma (proliferative arrest, differentiation, and senescence) [18, 19], and hepatocellular carcinoma (apoptosis, differentiation, senescence, and dormancy) [20]. 

In tumor dormancy, cells can restore their neoplastic properties upon MYC reactivation. In order to improve therapy, it is important to distinguish when MYC inactivation leads to complete tumor regression characterized by permanent loss of malignant phenotype and when it simply results in a reversible state of tumor dormancy [21, 22]. 

MYC inactivation in MYC-induced lymphoma leads to differentiation, apoptosis, and complete tumor regression. Therefore, a permanent loss of a neoplastic phenotype occurs upon MYC inactivation. In osteogenic sarcoma, MYC inactivation induces differentiation and proliferative arrest but does not induce significant apoptosis. MYC reactivation in these apparently differentiated cells either has no consequences or leads to apoptosis. Only in very rare cells is there restoration of neoplastic properties. In hepatocellular carcinoma, MYC inactivation leads to differentiation and then eventually to gradual apoptosis of most of the tumor cells. Upon reactivation of MYC, these differentiated cells quickly become tumorigenic [23].

Senescence is the growth-arrest process by which normal cells are restrained from malignant transformation. Oncogene inactivation-induced senescence (OIIS) is the irreversible cell cycle arrest of normal cells in response to inactivating an oncogene. We have recently shown cellular senescence resulting from MYC inactivation to depend on the host immune system [24]. Tumor regression upon inactivation of the MYC oncogene is associated with cellular senescence. Cellular senescence is an essential factor in bringing about sustained tumor regression upon MYC inactivation.

The *p53* gene has been shown to suppress tumor angiogenesis and regulate thrombospondin-1 (*TSP-1*), a potent antiangiogenic protein, expression [25]. The loss of p53 upon MYC inactivation leads to a deficit of TSP-1 and this inhibits angiogenesis thus impeding tumor regression. Restoration of p53 leads to sustained tumor regression upon MYC inactivation. Therefore, either p53 or TSP-1 is required upon MYC inactivation to shut down angiogenesis and induce sustained tumor regression [26].

Tumors undergo regression initially regardless of the status of the host immune system. But in hosts that are immune compromised, tumor elimination is incomplete and the tumors eventually relapse. An intact immune system is required for oncogene inactivation-induced senescence, inhibition of angiogenesis, and chemokine expression, which lead to sustained tumor regression. CD4+ (but not CD8+) T-cell deficiency was enough to impede sustained tumor regression. The secretion of TSP-1 is markedly decreased in immune compromised versus wildtype hosts. TSP-1 expression requires host immune cells particularly CD4+ T cells. Reconstitution of immune compromised mouse with CD8+ T cells still showed significant minimum residual disease, whereas reconstitution with CD4+ T cells showed no minimum residual disease, the same result found in wildtype hosts upon MYC inactivation. Hence, simply restoring CD4+ T cells was sufficient to eliminate minimum residual disease and to lead to sustained tumor regression. CD4+ T cells are the crucial host effector population necessary for tumor regression upon MYC inactivation. TSP-1 is important in immune effectors for sustained regression upon MYC inactivation, and overexpression of TSP-1 in immune compromised hosts is sufficient to increase the duration of sustained tumor regression upon MYC inactivation [27, 28].

### 2.2. Mathematical Model

Our mathematical model uses various modeling techniques that were each shown to be necessary to accurately recapitulate the experimental observations.

#### 2.2.1. Multiple Tumor Cell States

**Figure 1:**
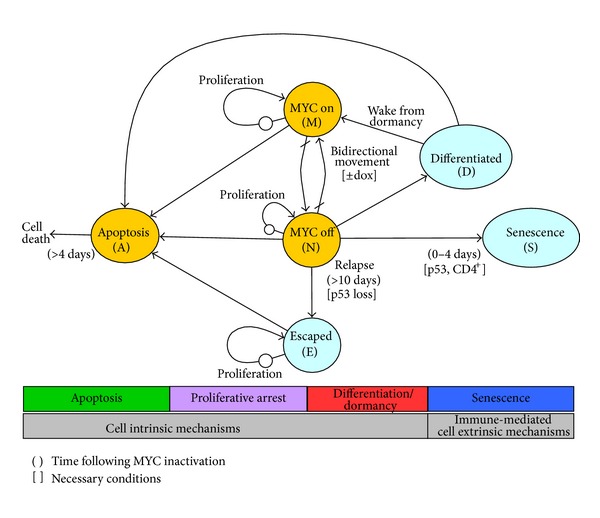
Mathematical model of cellular states. Former model shown in gold with additions shown in blue. The arrows with slashes corresponding to ±dox indicate that this is an independent variable controlled experimentally. The arrows representing proliferative loops have an implicit state during proliferation representing the mitotic phase of the cell cycle.

We created a new mathematical model (Figure 1) of tumor growth/regression kinetics incorporating cell intrinsic mechanisms (apoptosis, proliferative arrest, differentiation/dormancy) and immune-mediated cell extrinsic mechanisms (senescence). The stochastic model consists of 6 cellular states (“MYC on,” “MYC off,” apoptosis, proliferating, differentiated, senescent) with probabilistic transitions and the ability to control the expression of transgenic MYC using the tetracycline system. In our state transition model, discrete numbers of tumor cells move from one state to another, unlike other models where single classes of cells (e.g., tumor, immune, or normal) are modeled by single homogeneous states with no transitions between them. The core of the model consists of the “MYC on” and “MYC off” states, controlled in the conditional transgenic mouse model through doxycycline (dox) in the drinking water. This is central to the model since we are specifically investigating the effect of targeted therapeutics. “MYC off” tumor cells have been shown to be able to develop mechanisms to turn MYC back on without doxycycline through tTA, Notch, MAPK, or Wnt pathways and are represented by the “Escaped” node in the model [17]. Tumor cells may undergo proliferation or apoptosis, and “MYC off” tumor cells may alternatively undergo differentiation or oncogene inactivation-induced senescence.

The structure and topology of our mathematical model is based on *in vivo *observations from numerous studies [10, 17, 23, 24, 27]. We added an explicit transition in the model from “MYC off” to “Differentiated” to represent differentiation due to oncogene inactivation. We tested parameters over a number of values and chose values that most closely matched experimental data. Additionally, we added a transition from “Differentiated” back to “MYC on” to indicate that some tumor cells (e.g., hepatocellular carcinoma) that have differentiated to an apparently normal state may be dormant but possess the ability to regain neoplastic properties [23, 29]. The transition from “MYC off” to “Oncogene Inactivation Induced Senescence” is dependent on both p53 and CD4+ T cells and represents immune-mediated effects. 

Tumor cells can exist in one of six different states; the number of cells in each state is represented as follows. *M*, the number of cells in which MYC is “on”; *N*, cells in the MYC “off” state and still under the control of the tetracycline system; *A*, cells which have irreversibly committed to apoptosis; *D*, cells that have differentiated back into a quasinormal state, although in some tumor types they retain their neoplastic capability if MYC is reactivated; *S*, cells that have irreversibly committed to oncogene inactivation-induced senescence (OIIS); and *E*, cells that have escaped their addiction to MYC (e.g., through mutations in the tetracycline control elements or by activating expression of genes downstream of MYC). Note that due to pharmacokinetics and a number of other factors, the transitions between *M* and *N* are noninstantaneous and tetracycline dependent where the path is only open (nonzero) in one direction at a time. Note that because our current biological data uses mice that are either immunocompetent or immunodeficient (with no intermediate states and no direct measurements of immune effector cell populations), we do not explicitly model the immune cell numbers but rather have immune status dependent state transitions.

#### 2.2.2. Stochastic Transitions between Tumor Cell States

**Figure 2:**
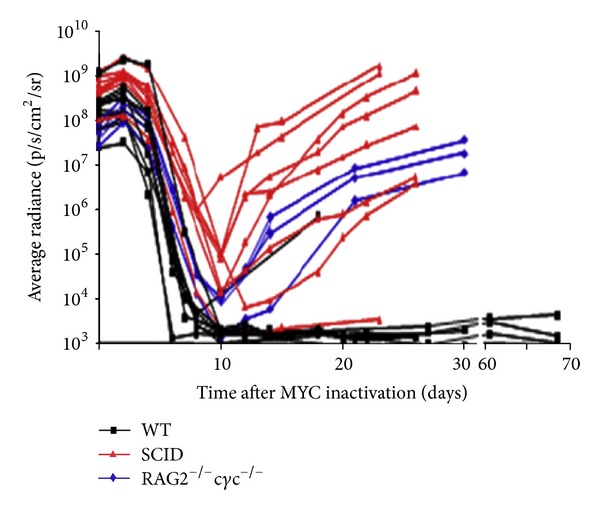
Tumor regression and relapse kinetics as measured by bioluminescence imaging. Wildtype (WT) are immunocompetent mice while SCID and RAG2^−/−^c*γ*c^−/−^ are immunodeficient mice. Excerpted from [27].

The experimental data shows the variability in relapse kinetics, which a deterministic model cannot capture. Hence, stochasticity was added to the model. We use random sampling from a multinomial distribution (well approximated by binomial due to very low pertime step probabilities) to represent a stochasticity in the number of cells transitioning from one state to another, enabling us to recapitulate the variability in tumor relapse (Figure 2). 

Some parameters of the model are immune system dependent, some are MYC expression dependent, and others are tumor type dependent (Table 1).

**Figure 3:**
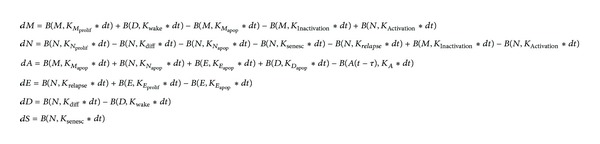
Set of equations represented by the model shown in Figure 1. *B*(*n*, *p*) is a binomial random variable of *n* the Bernoilli trials with probability of success *p*. *K* are rate constants independent of the time step size. Note that all the main variables are a function of *t* shortened for the sake of clarity. For example, *M* is *M*(*t*).

Figure 3 shows the governing equations for each of the 6 states. We integrated the equations using Euler's method with a time step (Δ*t*) of 0.02 days per iteration, which was much faster than any of the kinetic parameters. Model parameters are explained in Table 1.

#### 2.2.3. Delay Differential Equations

In biological processes, there are often “physical” delays making it vital to incorporate delays into the model in order to make the mathematical model closer to the real phenomenon. Examples of delay mathematical models in biology are population dynamics (e.g., Hutchinson's equation), ecology (e.g., the Lotka-Volterra predator prey), and immunology (e.g., delay in immune system response). We have implemented delay differential equations (on top of our stochastic framework) for the apoptosis state. There is a delay between when a cell commits to apoptosis and when cell death actually occurs. This is important since these cells that have committed to apoptosis (in state *A*) are still producing BLI signals, which are being measured. There is also a delay between when a cell commences mitosis and when daughter cells are actually produced, but we chose not to explicitly include this since at this time, the comparison to biological results is not significantly affected by whether or not we explicitly model the mitotic phase.

Although our modeling philosophy has been to create the simplest possible model that could explain the salient features of our experimental data, we found multiple tumor states and stochastic transitions without explicit delays to be insufficient to model some of our observations from bioluminescent imaging and immunohistochemistry. In particular, we found that proliferative arrest occurs almost immediately after oncogene inactivation but apoptosis was delayed for approximately 4-5 days. 

**Figure 4:**
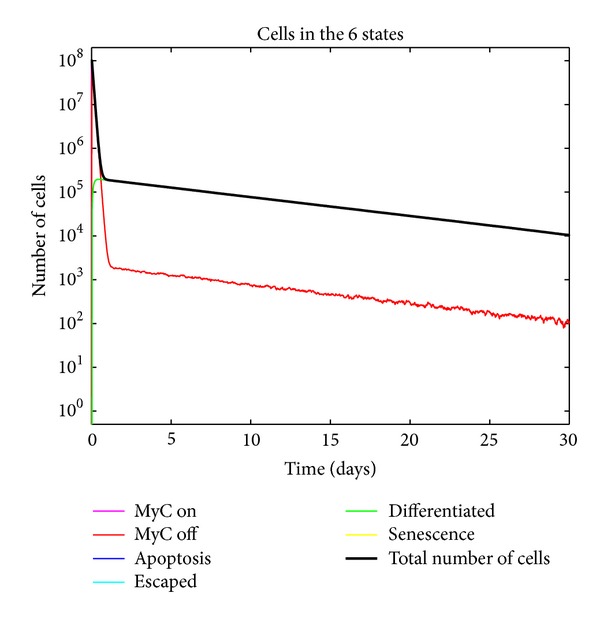
Simulated tumor growth kinetics with no delay and no escape of tumor cells from the conditional control of MYC due to an intact immune system. Note the lack of delay in the decrease of tumor cells as is observed in days 0–5.

Figure 4 shows the tumor growth kinetics in the absence of delay. This captures the necessity of adding delay to accurately represent the biology.

## 3. Results and Discussion

By running simulations, our model recapitulates features such as the different rates and delays in the tumor kinetics measured from *in vivo* experimental data from mouse models. Several emergent behaviors of the model have come to light. Empirically, proliferative arrest immediately follows oncogene inactivation but there is a 4-5 day delay in apoptosis. This was modeled by incorporating a delay between irreversible commitment to apoptosis and actual cell death. 

**Figure 5:**
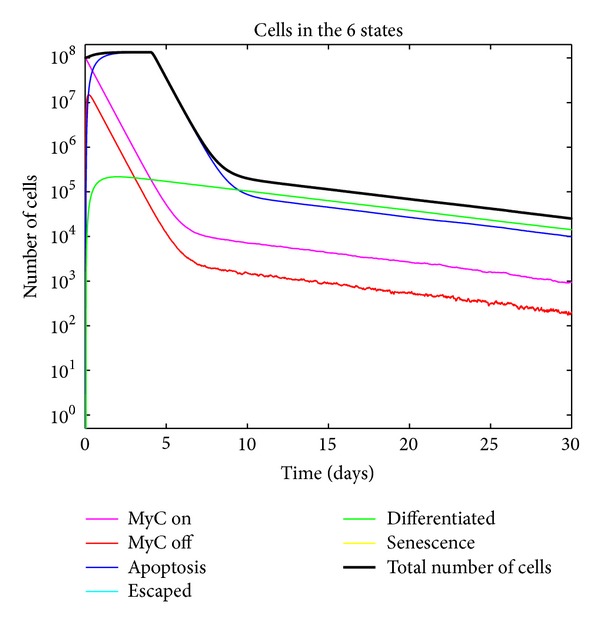
Simulated tumor growth kinetics with no escape of tumor cells from the conditional control of MYC due to an intact immune system. Note the correctly modeled delay in tumor cell death as is observed in Figure 2.

**Figure 6:**
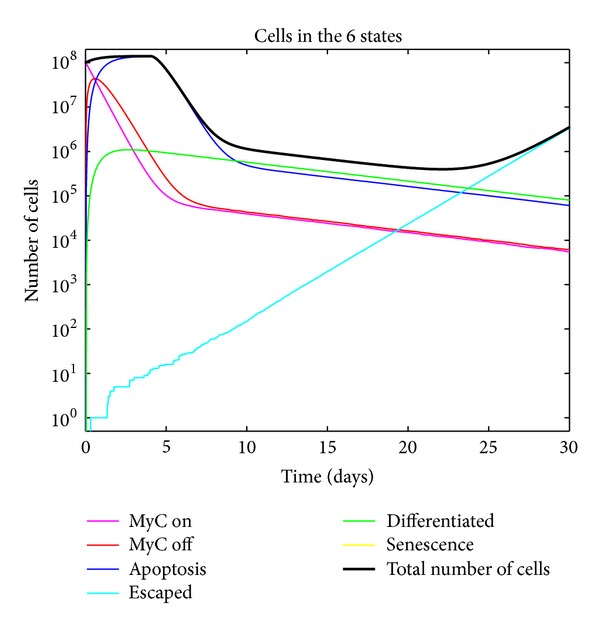
Simulated tumor growth kinetics with a low (but nonzero) rate of tumor cells escaping from conditional control of MYC due to a compromised immune system. Note the early growth kinetics of the escaped tumor cell population demonstrates stochastic variability that affects the timing of the relapse.

Furthermore, the rate of mutations leading to tumor relapse (which is captured in the term *K*_relapse_) had almost no bearing on the kinetics of the relapse. Instead, *K*_*E*-prolif_ dominates tumor relapse kinetics. We performed a basic simulation of regressing tumors followed by increasing the rate of tumor cells from “MYC off” to “Escaped” (increasing the term *K*_relapse_) that would have otherwise gone to “Oncogene Inactivation Induced Senescence.” The rate governing mutations leading to the transition from “MYC off” to “Escaped” had little effect on deterministic simulations, which were dominated by the growth rate of the escaped cells, but a significant effect on our stochastic model. The relapse of tumors due to absence of immune-mediated senescence is demonstrated in Figure 6. This indicates that the immune system plays a significant role in sustained tumor regression. From Figure 5, we see that in a WT mouse (which has an intact immune system), there is sustained tumor regression.

**Figure 7:**
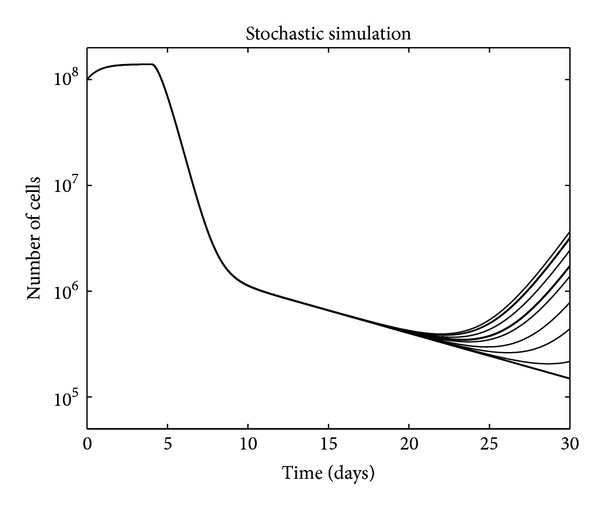
Variability in relapse kinetics captured using 20 stochastic simulations.

Empirically, the regression kinetics had little variability (in Figure 2, the curves all overlap) while the relapse growth kinetics showed greater variability (timing of the curves varies). Only adding stochasticity to our deterministic model can capture this variability in relapse in the different mouse models. The result from a run of 20 simulations is displayed in Figure 7.

## 4. Conclusions

While our model has yielded numerous hypotheses and insights about oncogene addiction, the model has various limitations. There are other important host-dependent factors that have yet to be added, namely, the inhibition of angiogenesis upon oncogene inactivation. Angiogenesis has a number of potential effects on portions of our model including growth rates, hypoxia-related resistance, and lymphocyte access to the tumor. Another limitation is that we do not currently model tumor stem cell properties. Many models have been developed that model the spectrum of stemness in tumor cells but we do not know how much effect this will have on our results. Similarly, other effects such as DNA methylation and transcription factor networks have not been included [30, 31]. Finally, our modeling of immune-dependent effects is done implicitly although we plan to add explicit model variables for immune effector cell populations that model tumor-immune interactions similar to predator-prey dynamics.

The initial results from our new model are helping to quantitatively hypothesize about the sequence of cellular mechanisms involved in tumor response to oncogene inactivation such as might be encountered using targeted therapeutics, and the interactions among them. These hypotheses will be tested experimentally using the same conditional control mouse models and *in vivo* bioluminescence imaging (and immunohistochemistry). We aim to eventually use this model to help to optimize multipronged treatment regimens for patients so as to defer or even eliminate tumor relapse. Avoiding immune destruction is a crucial hallmark because it has been shown that immune cells, CD4+ T cells in particular, are a required component for senescence, shutdown of angiogenesis and chemokine expression that result in sustained tumor regression [26, 27, 32–34].

This finding is captured in our model. If residual disease reaches a low enough level, relapse can be prevented. Large tumors have a great number of cells that can transition through an extremely low-likelihood event to the “Escaped” state. If the tumor is small enough, not enough cells remain to make it likely that any one will achieve the very low-likelihood “Escaped” state. Tumor burden is also an important factor in tumor regression mediated through the immune system. The immune system might not be able to attack and eliminate the tumor fast enough if the tumor burden is too high. Currently our model does not account for this but we are adding more sophisticated immune system components to the model in order to show this. Our model is simple in that all but one state (with explicit delay) have memoryless transitions and yet the model is able to recapitulate the complex response of tumors to oncogene inactivation. No age structuring of cells is required as with some other models [35, 36].

Our model offers more fidelity than models in the literature that just capture tumor cells in a single variable [37] because we are able to capture the various cellular states. These states have not yet been quantitatively captured over time *in vivo*. We are working on imaging methods to quantify distinct cellular processes such as apoptosis, and senescence, proliferation, which will eventually allow us to further validate our simulation results. Future work will include validating novel predictions from our model *in vivo*. 

## Figures and Tables

**Table 1:** Model parameters and values governing the response to oncogene inactivation.

Parameter	Estimated value (day^−1^)	Description
*K*_*N*_prolif_	0.1	Transition coefficient from the “MYC off” state to proliferating
*K*_diff_	0.02	Transition coefficient between “MYC off” state and differentiation state
*K*_*N*_apop_	2	Transition coefficient between “MYC off” and apoptosis state
*K*_senesc_	0.001	Transition coefficient between “MYC off” state and Oncogene inactivation induced senescence state
*K*_relapse_	0/3 × 10^−8^	Transition coefficient between “MYC off” state and escaped state (immunocompetent/immunodeficient)
*K*_wake_	0.1	Transition coefficient between differentiated state and “MYC on” state
*K*_*E*_prolif_	0.5	Transition coefficient from the escaped state to proliferating
*K*_*M*_apop_	0.05	Transition coefficient between “MYC on” and apoptosis state
*K*_*M*_prolif_	0.5	Transition coefficient from the “MYC on” state to proliferating
*K*_*E*_apop_	0.01	Transition coefficient between escaped state and apoptosis state
*K*_*D*_apop_	0.05/0.002/0.01	Transition coefficient between differentiated state and apoptosis state (tumor dependent: lymphoma, osteosarcoma, and hepatocellular carcinoma)
*K*_Inactivation_	0/2	Transition from “MYC on” to “MYC off” (depending on if MYC is on/off)
*K*_Activation_	2/0	Transition from “MYC off” to “MYC on” (depending on if MYC is on/off)
